# Investigating Associations Between Screen Time and Symptomatology in Individuals With Serious Mental Illness: Longitudinal Observational Study

**DOI:** 10.2196/23144

**Published:** 2021-03-10

**Authors:** Philip Henson, Elena Rodriguez-Villa, John Torous

**Affiliations:** 1 Mt Sinai School of Medicine New York, NY United States; 2 Beth Israel Deaconess Medical Center Harvard Medical School Boston, MA United States

**Keywords:** mHealth, schizophrenia, apps, mobile, screen time

## Abstract

**Background:**

Increasing screen time exposure from digital devices like smartphones has shown a variety of mixed associations with cognition, behavior, and well-being in adults and children but little is known about its associations with symptomatology in individuals with serious mental illness.

**Objective:**

To determine the range of associations between screen time and symptoms of individuals with mental illness, we utilized a method called specification curve analysis.

**Methods:**

In this observational study, we recruited smartphone-owning adults (≥18 years old) with schizophrenia and healthy controls. We installed 2 research-source smartphone apps, mindLAMP and Beiwe, to collect survey results, cognitive test results, and screen time metrics over a period of 3 months. Surveys were scheduled for twice a week, but participants were instructed to take the surveys naturally as much or as little as they wanted. Screen time was collected continuously in the background. A total of 140 participants was recruited from the outpatient clinic population as well as through general public advertising. Age-matched, smartphone-owning healthy controls were also part of the recruitment pool. A specification curve analysis was a priori designed to explore the relationship between every combination of independent variable and dependent variable in order to demonstrate to what degree screen time relates to symptoms in individuals with serious mental illness.

**Results:**

The sample consisted of 88 participants (54 with schizophrenia and 34 healthy controls) who completed both the initial and follow-up visits, completed at least one self-reported survey, and had a minimum passive data cutoff of 5 consecutive days. While we found an association between smartphone screen time metrics and cognition (adjusted R^2^=0.107, *P*<.001), specification curve analysis revealed a wide range of heterogenous associations with screen time from very negative to very positive. The effects differed based on diagnostic group, age bracket, type of regression model used, and the specific independent and dependent variables selected for analysis.

**Conclusions:**

The associations between screen time and mental health in patients with schizophrenia are heterogenous when examined with methods that reduce analytical bias. The heterogeneity in associations suggests that complex and personalized potential effects must be understood in the greater context of an individual. This analysis of longitudinally collected screen time data shows potential for future research that could benefit from high resolution metrics on smartphone use.

## Introduction

Technology pervades many, if not most, facets of daily life. Advances in functionality, speed, customization, and smart programming offer opportunities to access, communicate, and share resources with unprecedented efficiency. But this same connectivity has also raised issues around mental health impact. Digital devices are a mode for connectivity, explaining why 74% of Americans use a computer for their work [1] and 81% of Americans own a smartphone [2]. Screen time is thus a biproduct of productivity and sociability for many people. Resulting fears around increasing time spent looking at a screen and mental health concerns—whether it is for work or for leisure—have emerged.

Concerns, for youth especially, focus on how screen time may hinder physical activity, attention, cognition, and emotional well-being. Despite the vast increases in screen time, often across multiple devices and above recommended limits [3], data to validate or obviate concerns are limited. Studies to date show contradictory evidence on short-term and long-term effects of screen time on youth and adults [4]. Small sample sizes, self-report measures, and a deficit of longitudinal research have yielded inconclusive results. A recent paper used specification curve analysis (SCA) to highlight the degree to which study results measuring the impact of screen time on youth varied based on analytical choices [5]. The analysis enumerated the associations between screen time and emotional well-being among young people but also illustrated that the effect is smaller than other influences such as bullying.

Studies involving screen time among individuals with severe mental illness are even more scarce. On one hand, screen time may be of benefit for patients with serious mental illness as it may facilitate beneficial social connections that may be lacking offline and offer patients a sense of community and connection [6]. On the other hand, screen time may distract patients with serious mental illness from real-world obligations or expose them to harmful and stigmatizing content. One study in individuals with schizophrenia showed a positive correlation between phone use and functional capacity and cognition [7], but overall research on the topic is lacking. The need for understanding on this subject is critical, as research from a 2016 study suggests that nearly 50% of people with serious mental illness may spend up to 3 hours per day in front of their phone screen and nearly 20% may spend up to 10 hours per day on a computer screen [8], potentially making screen time a key exposure in their routine and daily life.

Screen time and exposure have likely only increased for all people, including those with serious mental illness, in the last 4 years since that 2016 study. Understanding how screen time has a positive or negative effect—or any effect at all—on the well-being of individuals with serious mental illness like schizophrenia is thus critical for ensuring today’s care remains responsive to the exposure and realities faced by patients. The data needed to identify problematic screen time involve total screen time, session time, and number of checks from smartphones. A recent study found that typical smartphone usage is relatively consistent and can be inferred with just 5 days of data [9]. On the other hand, habitual checking behaviors (sessions lasting less than 15 seconds) that may be indicative of preoccupation with mobile phones, can be inferred with just 2 days of data. These results, along with the finding that self-reported smartphone usage did not correlate with the objective measurements, suggest an important opportunity to use smartphone-derived screen time metrics in studying its effects.

Our research aimed to address a gap in the literature and understanding by investigating the effect of screen time on individuals with schizophrenia. Our unique dataset, with longitudinal objective screen time measures, self-report surveys, and both baseline and longitudinal cognition tests, offers an opportunity to begin to appreciate the impact of screen time on a subset of patients with serious mental illness. In relying on objective metrics of screen time derived from longitudinal phone screen on/off sensor data instead of single time-point, self-reported screen time, we hoped to avoid biases that have made prior works on screen time and mental health difficult to generalize. In this paper, we aimed to (1) investigate the association between screen time and baseline cognition in individuals with schizophrenia, (2) determine the impact of screen time on symptomatology in both people with schizophrenia and healthy controls via SCA to determine if effects hold across all possible analytical combinations, and (3) identify the association between screen time and symptomatology on an individual basis in both people with schizophrenia and healthy controls. We sought to determine the extent of screen time’s effect on symptoms in individuals with schizophrenia at both group and individual levels and expected to find complicated and heterogeneous associations between screen time metrics and symptomatology.

## Methods

### Longitudinal Data Collection Platforms

Two types of longitudinal data were collected: (1) active data in the form of participant self-reported surveys and cognitive tests and (2) passive data that included GPS, accelerometer, and screen time. Two research applications, mindLAMP and Beiwe, were installed on participants’ smartphones after receiving institutional review board approval at the Beth Israel Deaconess Medical Center [10,11].

### Participants

For both studies, smartphone-owning adults (≥18 years old) were recruited from the greater Boston area starting August 2018 through the Massachusetts Mental Health Center in Boston, MA and general public advertising for convenience sampling of controls. A total of 140 participants enrolled after signing written informed consent, 6 dropped out, and 46 were excluded for not providing at least one self-reported survey or having inadequate screen time data (a minimum of 5 days of smartphone usage was used as a passive data cutoff). Of the 88 remaining participants, 54 had a clinical diagnosis of schizophrenia (SZ), and 34 were healthy controls (HC). All participants owned a smartphone and were given a smartwatch for the duration of the study to assist in data collection. Demographic information can be found in Table 1.

**Table 1:** Demographic characteristics of 88 smartphone-owning participants from the greater Boston area.

Characteristics		HC^a^ (n=34)	SZ^b^ (n=54)	*P* value
Age (years), mean (SD)		39.62 (14.56)	33.02 (11.71)	.250
**Gender, n (%)**				.681
	Male	19 (56)	25 (46)	
	Female	13 (38)	25 (46)	
	Other	2 (6)	4 (7)	
**Race, n (%)**				<.001
	American Indian or Alaskan Native	0 (0)	4 (7)	
	Asian	27 (79)	3 (6)	
	Black or African American	2 (6)	19 (35)	
	Multiracial or other	1 (3)	1 (2)	
	Native Hawaiian or Pacific Islander	0 (0)	1 (2)	
	White Caucasian	4 (12)	20 (37)	
	Not reported	0 (0)	6 (11)	
**Education, n (%)**				<.001
	Some high school	0 (0)	2 (4)	
	High school graduate or GED^c^	3 (9)	15 (28)	
	Some college	2 (6)	20 (37)	
	4-year college graduate or higher	29 (85)	17 (32)	

^a^HC: healthy control.

^b^SZ: clinical diagnosis of schizophrenia.

^c^GED: General Educational Development test.

### Data Collection Protocol

After signing informed consent, participants completed paper-and-pencil symptom surveys, completed a cognitive assessment with a validated iPad version of the Brief Assessment of Cognition in Schizophrenia (BACS, SZ group only) [12], and installed mindLAMP and Beiwe on their smartphones. BACS was not administered for the HC group due to the lack of a psychiatric diagnosis as well as the assessment’s specificity for individuals with schizophrenia. For 3 months, participants were notified on their smartphones to take 10 surveys per week: 2 each of mood (PHQ-9 [Patient Health Questionnaire-9]) [13], anxiety (GAD-7 [7-item Generalized Anxiety Disorder assessment]) [14], sleep, and sociability. Each survey ended with a cognitive test: Jewels A or Jewels B, which are smartphone-adapted versions of the classic Trails-A and Trails-B tasks to assess a wide variety of cognitive domains including attention, visual search, task switching, and psychomotor speed [15]. Jewels B was used for the analysis as it is a more complex task and has shown better separation between individuals with psychosis and healthy controls [16]. A single score, or “beta value,” was used to represent performance on a Jewels task and takes into account both accuracy and error rate. Meanwhile, the Beiwe app collected multiple passive data streams (GPS, accelerometer, screen on/off, and call and text logs) simultaneously and uploaded the data to a Health Insurance Portability and Accountability Act–compliant server every hour. Raw screen time (in seconds) was calculated by summing the intervals between “Screen On” and “Screen Off” data points. While participants were paid for their clinical visits and in-person surveys, no study compensation was provided for app engagement or survey completion.

### Data Analysis

All analyses were performed using the R programming language (version 3.6.2 [17]). Raw screen time data were aggregated by day and processed into 3 main screen time metrics: (1) screen time (seconds), (2) session time (seconds), and (3) number of checks (unitless). Session time was calculated by dividing screen time by the number of sessions and checks (ie, “habitual checking behaviors” were sessions lasting less than 15 seconds) [9]. Smartphone surveys were also aggregated by day, and survey scores were averaged if more than one of the same survey was taken that day (eg, separate PHQ-9 results of 10 and 11 on the same day would be converted to 10.5).

Correlations between the first month of screen time and baseline cognition were conducted using the Spearman rank correlation coefficient, and *P* values were adjusted using the false discovery rate method. Multivariate multiple linear regression was performed on longitudinal screen time and Jewels B cognition beta values.

SCA was inspired by Orben and Przybylski [5] and aided by the “specr” package in R [18]. Gender (male or female) was added as a covariate, and 2 models were used: linear model and generalized linear model. Groups were separated for SCA based on age (<30 years old or ≥30 years old) and diagnostic group (SZ or HC).

Linear model regression was performed at the individual level between screen time and symptoms, and regression estimates were arranged from high to low. Only individuals with survey results in each of the 4 survey categories of interest (mood, anxiety, sleep, social) were included in this portion of the analysis.

## Results

### Cognition

Participants with schizophrenia (n=54) were assessed at baseline for cognition via BACS, and all 88 participants (54 SZ and 34 HC) were assessed longitudinally via the Jewels B assessment within the mindLAMP smartphone app.

#### Baseline

Among the 6 subdomains of the BACS (Verbal Memory, Verbal Fluency, Digit Sequencing, Symbol Coding, Token Motor, and Tower of London), Spearman correlations between screen time metrics and SZ baseline BACS subdomains ranged from –0.17 to 0.29, but there were no significant correlations (*P* values ranged from .25 to .98).

#### Longitudinal

Multivariate multiple linear regression revealed a significant regression equation in SZ for the association of the screen time metrics (number of sessions, number of checks, screen time, and session time) with longitudinal beta values for cognition (Jewels B assessment [16]): *F*_4,144_=5.43, *P*<.001 with an adjusted R^2^ of 0.107. The beta values used to represent cognition take into account accuracy and error rate, and the greater the beta value, the better the performance. The regression equation for HC was not significant (adjusted R^2^=0.035, *P*=.068).

### Specification Curve Analysis

**Figure 1:**
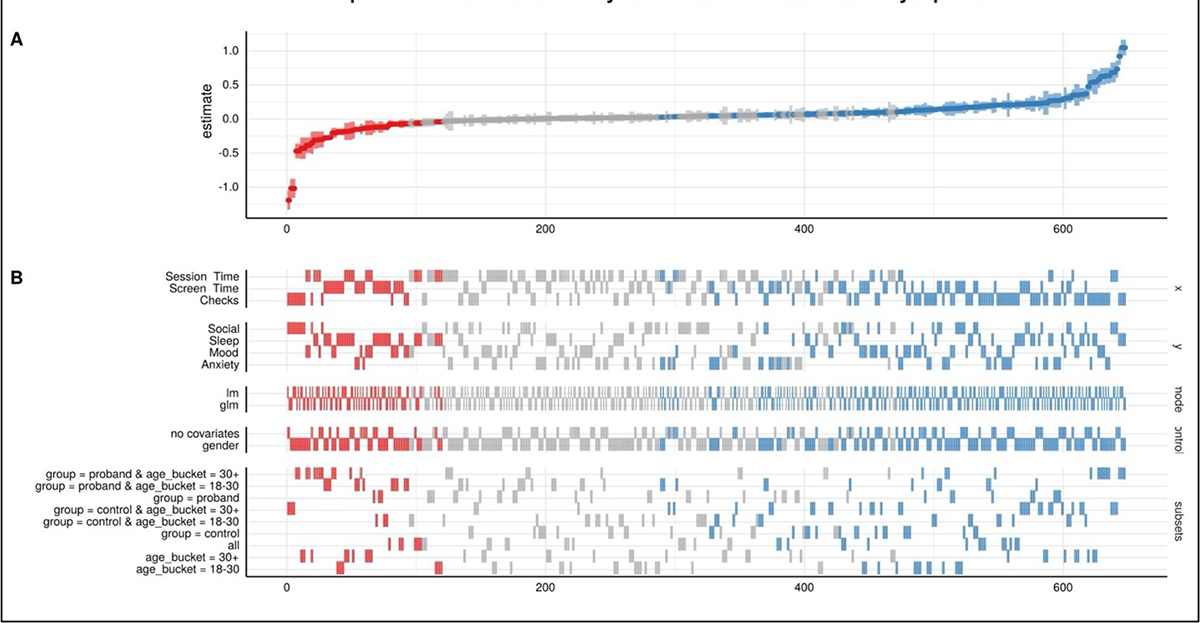
Specification curve analysis of screen time and symptoms, as a visual representation of the >600 combinations of regression analyses between screen time metrics and self-reported mood, anxiety, sleep, and sociability: (A) regression coefficient, (B) variables.

SCA on data for all 88 participants (54 SZ and 34 HC) revealed estimates (regression coefficient *β*) for over 600 combinations, or specifications, ranging from –1.19 to 1.05 (Figure 1). The figure is meant to be a high-level representation of the heterogeneity of associations between screen time metrics and symptoms, displayed in order of most negative on the left to most positive on the right. If we were to zoom in, we could see, for example, that an individual column within the red area might involve screen time, sociability, linear model, covarying for gender, and including participants with schizophrenia over the age of 30 years. For that group, the model found a significant negative association between self-reported sociability behavior and smartphone screen time. Individual analyses can be read as a vertical column, with each column (ie, specification) representing a unique combination of variables that was tested in this analysis. For example, the leftmost column of results involves checks, social, linear model, no covariates, and the control group of participants aged ≥30 years. In other words, this is the most negative association (*β*=–1.19) and suggests that more checking behavior was associated with worse reported sociability for an older control group while not adjusting for gender using a linear model. On the right side of the plot, the most positive association (*β*=1.05) was between checks and sleep (ie, more checking behavior was associated with better reported sleep) in SZ, but not HC, for individuals over 30 years old using a generalized linear model and adjusting for gender.

### Individual Participant Regression

**Figure 2:**
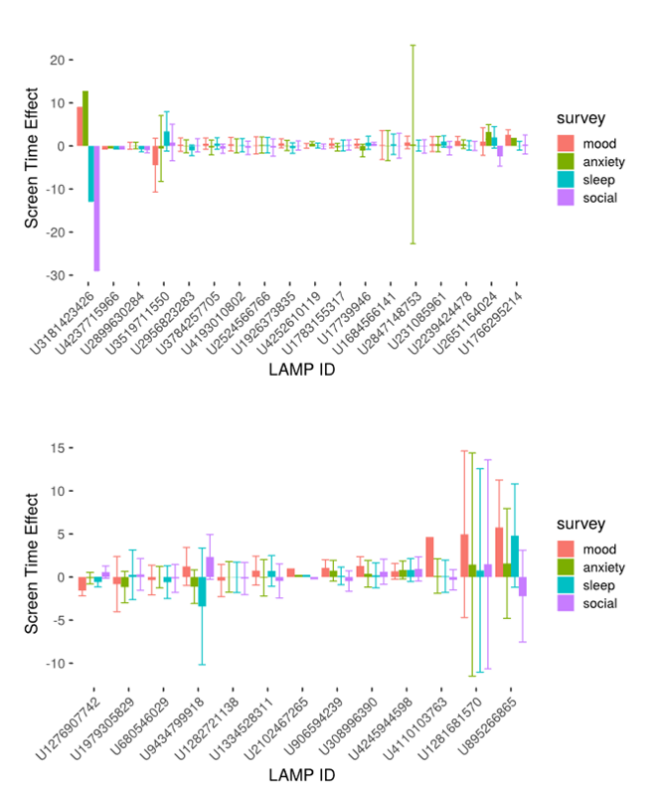
Individual effects of screen time on symptoms measured using the mindLAMP mobile phone app, plotted from low to high for (A) participants with a clinical diagnosis of schizophrenia (n=18) and (B) healthy controls (n=13), with self-reported survey scores in all categories.

As shown in Figure 2, linear model estimates at the participant level revealed differences in screen time’s effect on symptoms among individuals as well as between groups, with lower and more negative values identified in the SZ group (mean –0.136, SD 4.3) than in the HC group (mean 0.51, SD 1.6). Individual estimates ranged in SZ from –28.9 for sociability to 12.6 for anxiety (mean 0.51, SD 1.6) and in HC from –3.4 to 5.8 in HC (mean –0.136, SD 4.3). Note that only participants with results in all 4 survey domains were included in this analysis (18 SZ and 13 HC), and completion of surveys was optional in the study.

## Discussion

### Principal Findings

Using longitudinal and objective measurements related to screen time, we found a range of positive and negative associations with mental health symptoms at both population and individual levels in patients with schizophrenia. We found no significant associations between screen time and baseline cognition but did find associations in schizophrenia between cognition as measured by the Jewels B cognitive task and screen time metrics (adjusted R^2^=0.107, *P<*.001). The low R^2^ indicates that, despite the significant trend, much of the variability cannot be explained by the model. Nonetheless, patterns of smartphone use may be related to underlying cognitive functioning—presenting opportunities for capturing novel data and designing more engaging apps.

However, it is important to consider the multitude of factors in addition to screen time that may contribute to well-being, symptomatology, and cognition. An SCA on 3 large data sets of over 350,000 people found that many other personal and behavioral factors can have as much, if not more, of an effect on well-being than technology use [5]. For example, bullying and marijuana use had a greater negative effect on well-being than technology use, whereas proper sleep and nutrition had a greater positive effect. The authors even drew attention to neutral factors like eating potatoes as having nearly as negative of an effect on technology use. Our dataset is much smaller but does rely on objective metrics of screen time and also suggests that at a population level, screen time itself is not highly associated with symptomology.

As with previous screen time analysis studies, our results show a wide range of regression results based on the chosen analysis and isolated components. However, the SCA did bring to light some interesting results around group differences in the study. For example, both screen time and checking behavior had greater positive associations with symptoms of older controls than younger controls (x2.1 and x36.9, respectively) and checking time across all ages in the SZ group had a more positive association with symptoms than in the HC group (x3.48). While there are many potential reasons for these associations beyond the scope of this study, they highlight potential beneficial associations contrary to some perceptions that older adults or those with schizophrenia may not want to engage or even use technology. Results that screen time and checking behaviors were associated with improved sleep outcomes in those with schizophrenia but not controls also highlights that results in healthy controls do not always mirror those in patients, and that caution is necessary if seeking to apply the broader research base of screen time in the general population to those with serious mental illness.

On an individual basis, our results suggest that simple rules or guidance around screen time and mental health for individuals with schizophrenia, or controls, may not be practical. The individual participant analysis revealed heterogeneity in the effect of screen time on symptoms, with individual screen time effect estimates ranging in the SZ group from –28.9 for sociability to 12.6 for anxiety (mean 0.51, SD 1.6) and from –3.4 to 5.8 in the HC group (mean –0.136, SD 4.3). In this sample, the effects on the HC group were more positive but less variable than in the SZ group. This could be because the controls in this smartphone study may be naturally more interested in technology and thus more technology-literate and use their smartphones more regularly in ways that improve symptoms, for example playing a game to reduce stress. In addition, the greater variability in the SZ group may be due to inherent variability in symptoms and behavior associated with serious mental illness that is not present in healthy controls.

There are very few studies investigating neurocognitive effects of screen time in schizophrenia, and our results do not yet suggest conclusive evidence to fill this literature gap. Our baseline assessment of cognition was not associated with any screen time metrics, but this analysis was performed at a population level, and results may be washing out due to individual variance. There is, however, evidence that past mobile phone use in older (>40 years old) individuals with schizophrenia is associated with higher global cognitive performance [7]. Despite the mean age difference, this is in line with our longitudinal assessment finding of an association between phone checking behavior and higher performance on the Jewels B cognitive task.

While several individual studies have found associations between screen time and symptoms of depression or anxiety in both adults and children [19-21], our SCA results are in agreement with a recent large dataset analysis (n=355,358) suggesting a complex relationship between screen time and symptoms [5]. We did not have data on other personal and behavior factors (eg, bullying, nutrition) for comparison, but the range of estimates produced by the SCA analysis demonstrates that the effect of screen time is still heterogenous and difficult to parse given all the variables.

### Limitations

As with all studies, there are several limitations that need to be addressed. First, with longitudinal behavioral data, it is important to note that behavior and symptomatology can change throughout the day, so while daily aggregation employed in our study represents a high resolution for longitudinal analysis, it could be higher to capture fluctuations within the day. Still, compared to survey studies where patients estimate their mental health and screen time over weeks or even months, our methods offer improvement. Second, individual analysis only included participants with data in all survey categories. We are likely missing information from those less engaged with the app or who elected to take only a subset of surveys due to the naturalistic study design. Third, there may be a time lag between screen time and symptom change that is not accounted for as independent and dependent variables are paired for a given day, although such an effect has not yet been well characterized in the research to date. Finally, while controls were matched on age, they differed in education and race, potentially due to convenience sampling, which may have had a confounding effect.

### Conclusion

Increasing screen time is a growing concern, and despite recent research efforts, there are very few studies reporting the effect of screen time on individuals with schizophrenia. The importance of a priori analysis and transparent methods around digital mental health is also highlighted in our results, which show how divergent conclusions can be supported if using more limited analysis. Our results show that variance at the individual and population levels can account for drastically different reporting of screen time’s associations with symptoms, from very negative to very positive, demonstrating a complex relationship that requires further exploration.

## Abbreviations

BACS: Brief Assessment of Cognition in Schizophrenia
GAD-7: 7-item Generalized Anxiety Disorder assessment
HC: healthy control
PHQ-9: Patient Health Questionnaire-9
SCA: specification curve analysis
SZ: clinical diagnosis of schizophrenia

## References

[1] Mamedova S, Pawlowski E (2018). A Description of U.S. Adults Who Are Not Digitally Literate. U.S. Department of Education.

[2] Mobile FS (2019). Mobile Fact Sheet. Pew Research Center.

[3] Fakhouri THI, Hughes JP, Brody DJ, Kit BK, Ogden CL (2013). Physical activity and screen-time viewing among elementary school-aged children in the United States from 2009 to 2010. JAMA Pediatr.

[4] Stiglic N, Viner RM (2019). Effects of screentime on the health and well-being of children and adolescents: a systematic review of reviews. BMJ Open.

[5] Orben A, Przybylski AK (2019). The association between adolescent well-being and digital technology use. Nat Hum Behav.

[6] Naslund JA, Bondre A, Torous J, Aschbrenner KA (2020). Social Media and Mental Health: Benefits, Risks, and Opportunities for Research and Practice. J Technol Behav Sci.

[7] Depp CA, Harmell AL, Vahia IV, Mausbach BT (2016). Neurocognitive and functional correlates of mobile phone use in middle-aged and older patients with schizophrenia. Aging Ment Health.

[8] Gay K, Torous J, Joseph A, Pandya A, Duckworth K (2016). Digital Technology Use Among Individuals with Schizophrenia: Results of an Online Survey. JMIR Ment Health.

[9] Wilcockson TDW, Ellis DA, Shaw H (2018). Determining Typical Smartphone Usage: What Data Do We Need?. Cyberpsychol Behav Soc Netw.

[10] Torous J, Wisniewski H, Bird B, Carpenter E, David G, Elejalde E, Fulford D, Guimond S, Hays R, Henson P, Hoffman L, Lim C, Menon M, Noel V, Pearson J, Peterson R, Susheela A, Troy H, Vaidyam A, Weizenbaum E, Naslund JA, Keshavan M (2019). Creating a Digital Health Smartphone App and Digital Phenotyping Platform for Mental Health and Diverse Healthcare Needs: an Interdisciplinary and Collaborative Approach. J. technol. behav. sci.

[11] Torous J, Kiang MV, Lorme J, Onnela J (2016). New Tools for New Research in Psychiatry: A Scalable and Customizable Platform to Empower Data Driven Smartphone Research. JMIR Ment Health.

[12] Atkins AS, Tseng T, Vaughan A, Twamley EW, Harvey P, Patterson T, Narasimhan M, Keefe RSE (2017). Validation of the tablet-administered Brief Assessment of Cognition (BAC App). Schizophr Res.

[13] Kroenke K, Spitzer RL, Williams JBW (2001). The PHQ-9: validity of a brief depression severity measure. J Gen Intern Med.

[14] Löwe B, Decker O, Müller S, Brähler E, Schellberg D, Herzog W, Herzberg PY (2008). Validation and standardization of the Generalized Anxiety Disorder Screener (GAD-7) in the general population. Med Care.

[15] Salthouse TA (2011). What cognitive abilities are involved in trail-making performance?. Intelligence.

[16] Liu G, Henson P, Keshavan M, Pekka-Onnela J, Torous J (2019). Assessing the potential of longitudinal smartphone based cognitive assessment in schizophrenia: A naturalistic pilot study. Schizophr Res Cogn.

[17] (2021). The R Project for Statistical Computing. The R Foundation.

[18] Masur P, Scharkow M (2020). specr: Statistical functions for conducting specification curve analyses. GitHub.

[19] Madhav K, Sherchand SP, Sherchan S (2017). Association between screen time and depression among US adults. Prev Med Rep.

[20] Maras D, Flament MF, Murray M, Buchholz A, Henderson KA, Obeid N, Goldfield GS (2015). Screen time is associated with depression and anxiety in Canadian youth. Prev Med.

[21] Kremer P, Elshaug C, Leslie E, Toumbourou JW, Patton GC, Williams J (2014). Physical activity, leisure-time screen use and depression among children and young adolescents. J Sci Med Sport.

